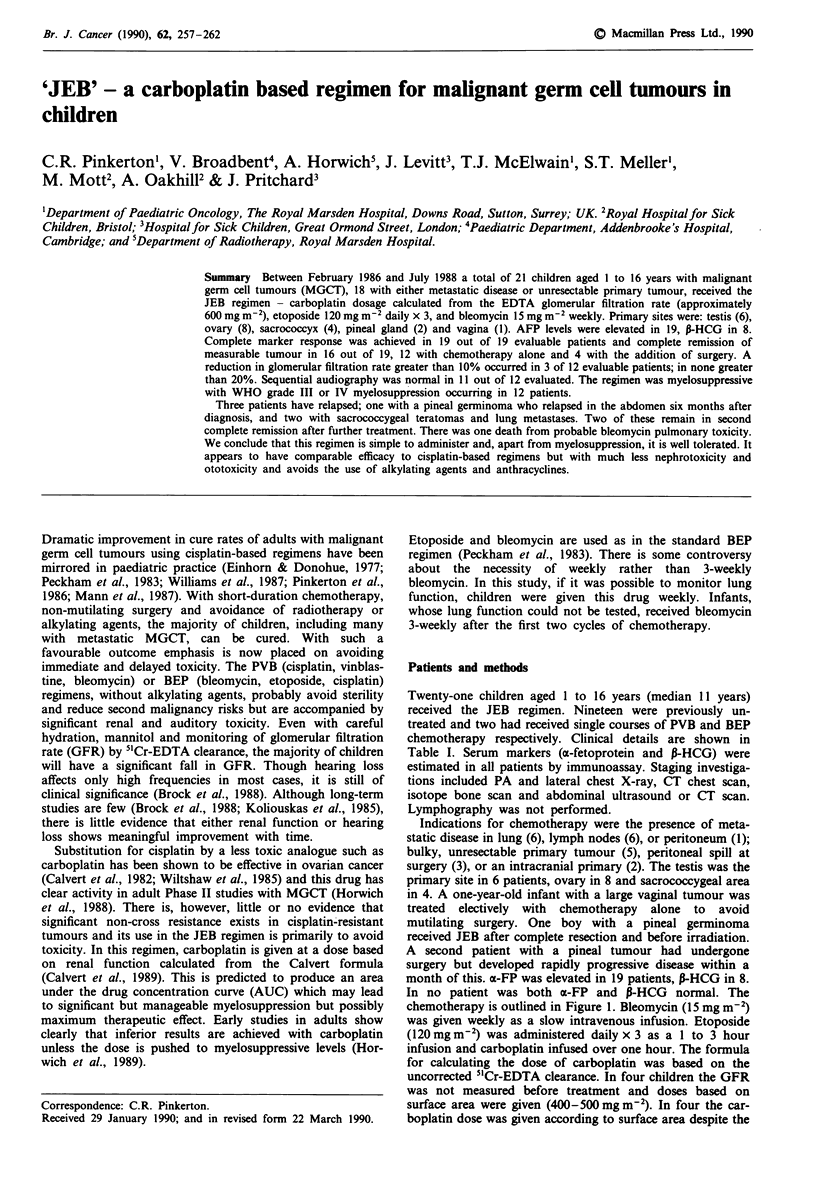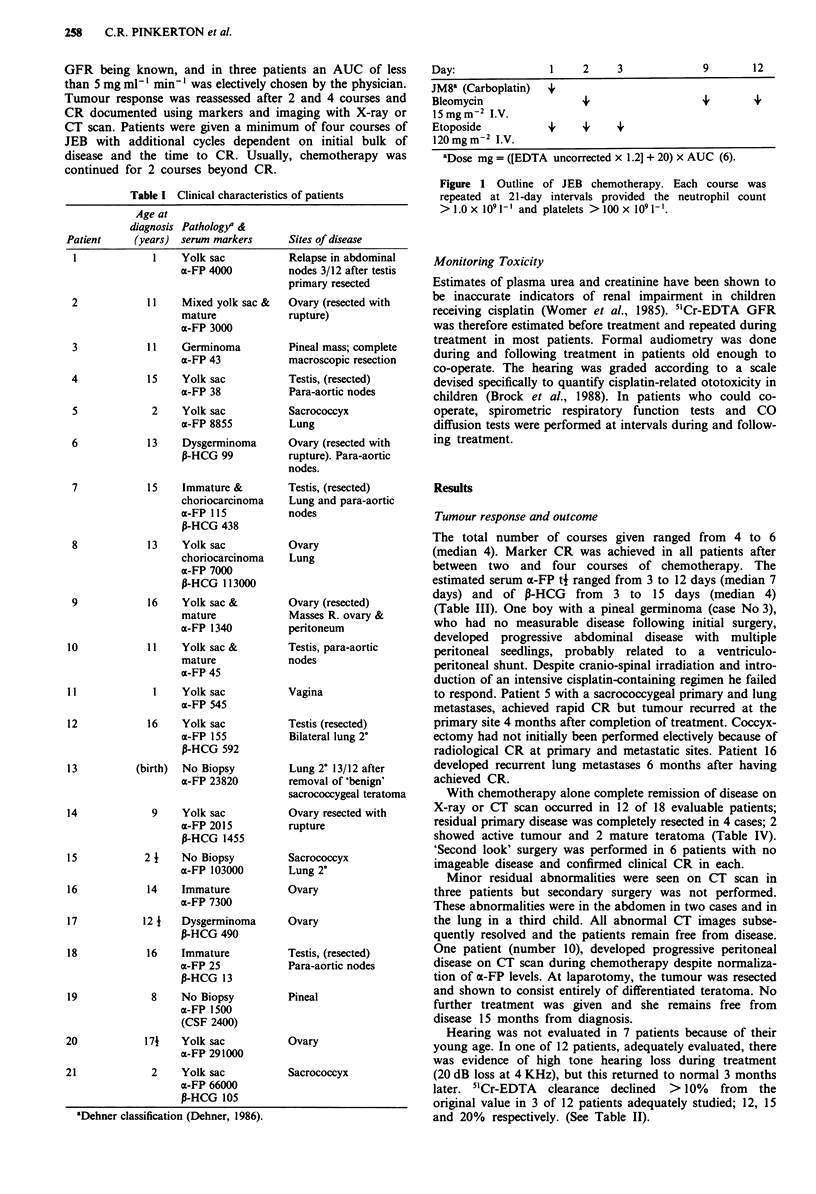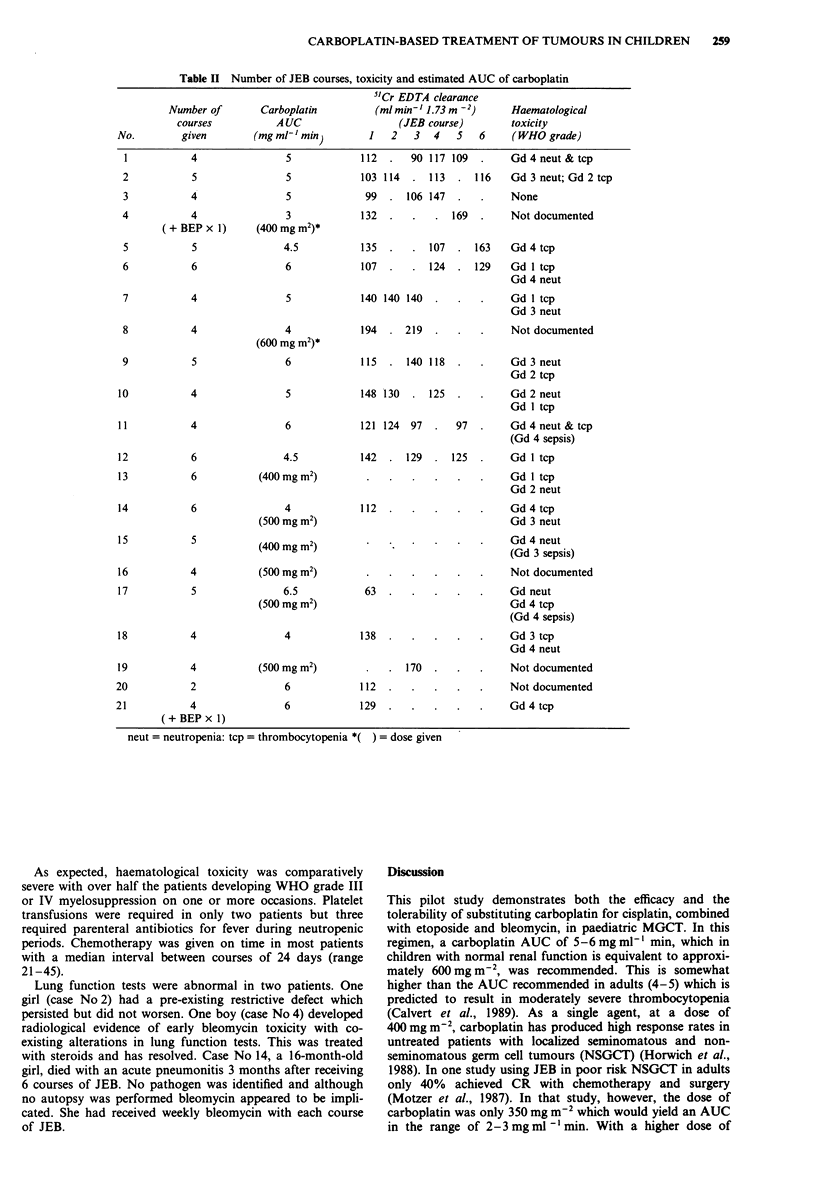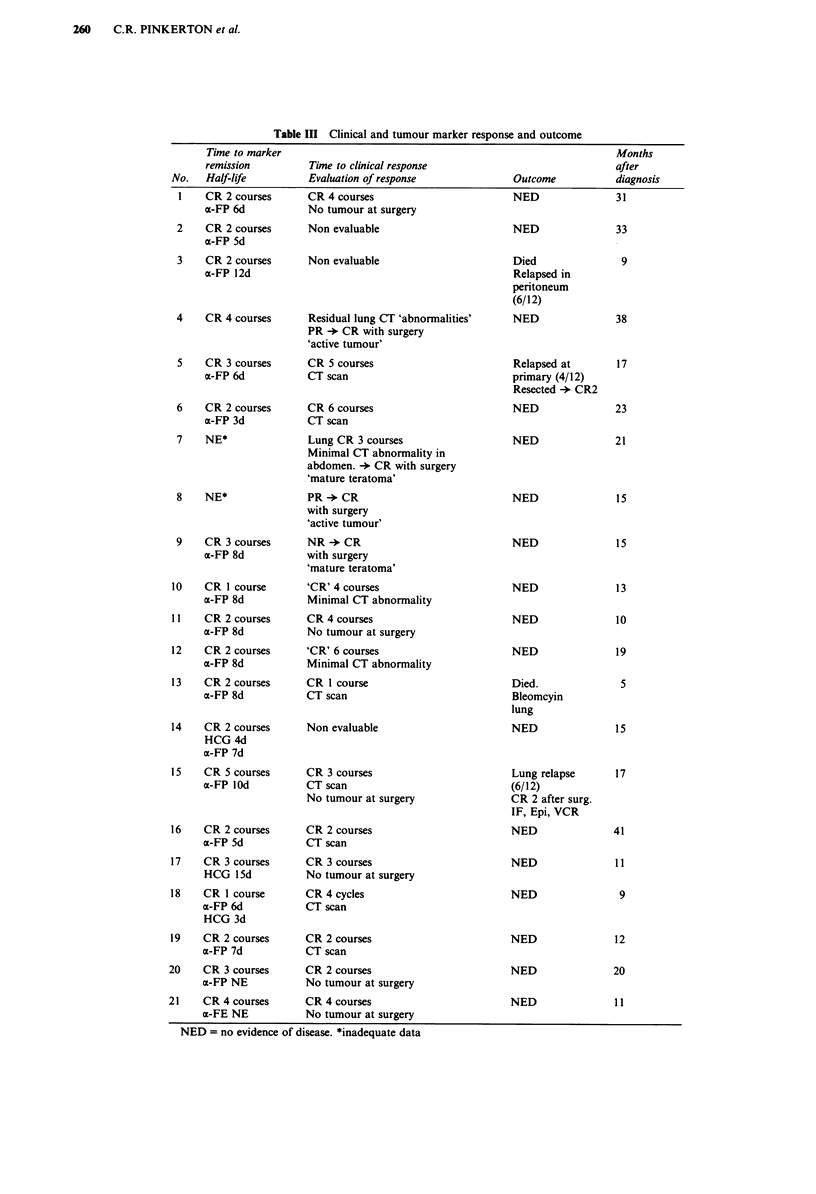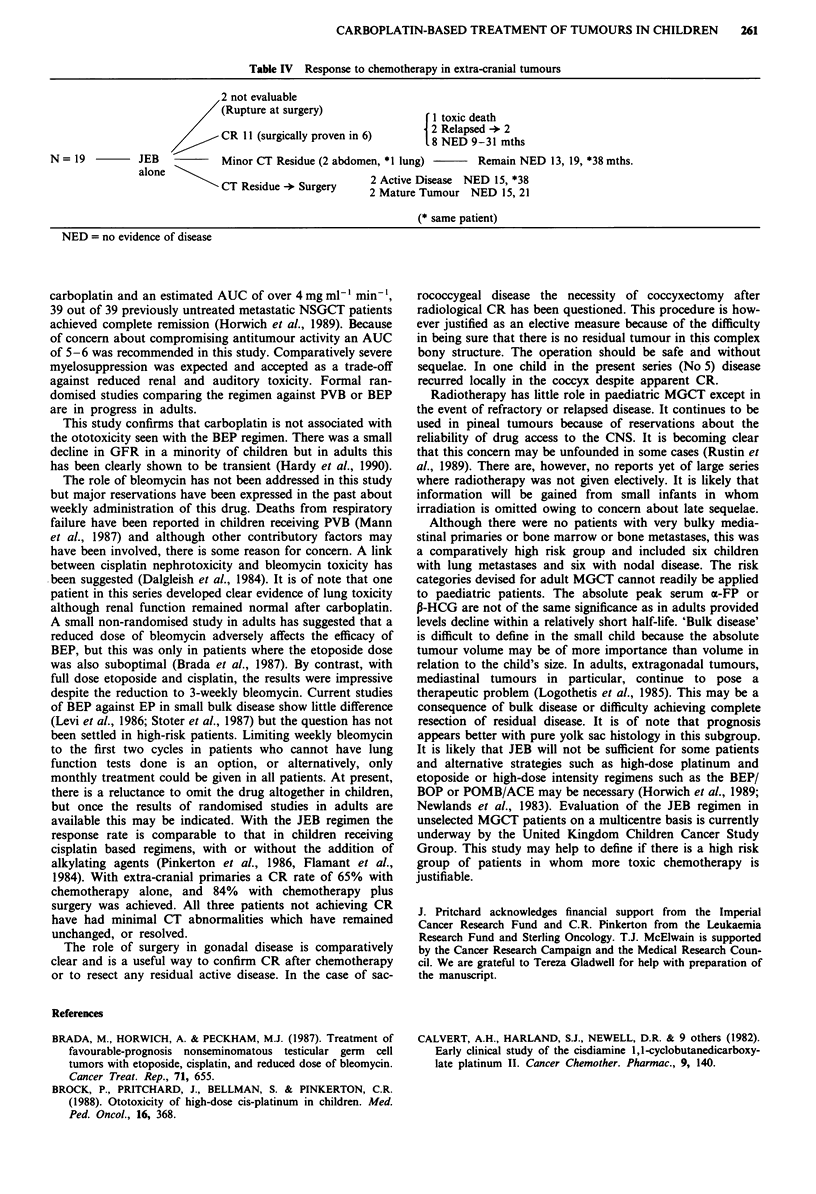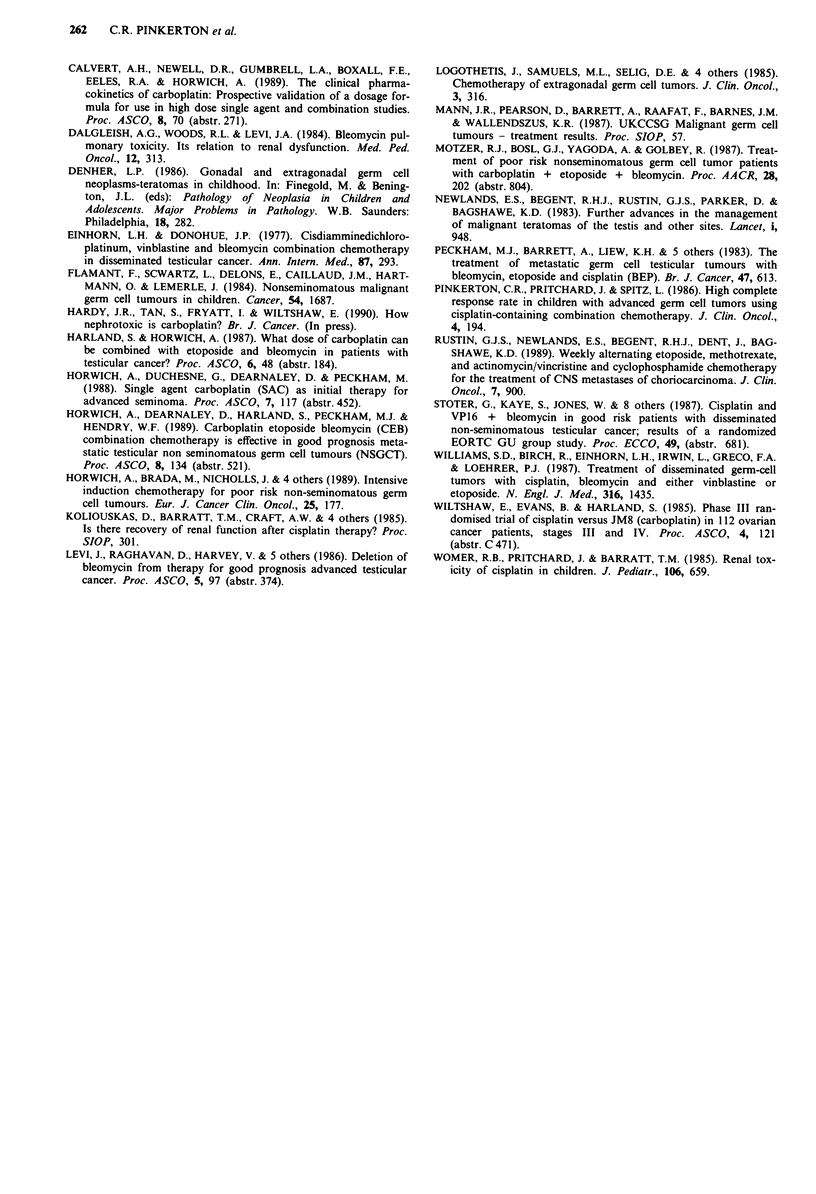# 'JEB'--a carboplatin based regimen for malignant germ cell tumours in children.

**DOI:** 10.1038/bjc.1990.272

**Published:** 1990-08

**Authors:** C. R. Pinkerton, V. Broadbent, A. Horwich, J. Levitt, T. J. McElwain, S. T. Meller, M. Mott, A. Oakhill, J. Pritchard

**Affiliations:** Department of Paediatric Oncology, Royal Marsden Hospital, Sutton, Surrey, UK.

## Abstract

Between February 1986 and July 1988 a total of 21 children aged 1 to 16 years with malignant germ cell tumours (MGCT), 18 with either metastatic disease or unresectable primary tumour, received the JEB regimen - carboplatin dosage calculated from the EDTA glomerular filtration rate (approximately 600 mg m-2), etoposide 120 mg m-2 daily x 3, and bleomycin 15 mg m-2 weekly. Primary sites were: testis (6), ovary (8), sacrococcyx (4), pineal gland (2) and vagina (1). AFP levels were elevated in 19, beta-HCG in 8. Complete marker response was achieved in 19 out of 19 evaluable patients and complete remission of measurable tumour in 16 out of 19, 12 with chemotherapy alone and 4 with the addition of surgery. A reduction in glomerular filtration rate greater than 10% occurred in 3 of 12 evaluable patients; in none greater than 20%. Sequential audiography was normal in 11 out of 12 evaluated. The regimen was myelosuppressive with WHO grade III or IV myelosuppression occurring in 12 patients. Three patients have relapsed; one with a pineal germinoma who relapsed in the abdomen six months after diagnosis, and two with sacrococcygeal teratomas and lung metastases. Two of these remain in second complete remission after further treatment. There was one death from probable bleomycin pulmonary toxicity. We conclude that this regimen is simple to administer and, apart from myelosuppression, it is well tolerated. It appears to have comparable efficacy to cisplatin-based regimens but with much less nephrotoxicity and ototoxicity and avoids the use of alkylating agents and anthracyclines.


Br. J. Cancer (1990), 62, 257-262 © Macmillan Press Ltd., 1990

### 'JEB' – a carboplatin based regimen for malignant germ cell tumours in children

C.R. Pinkerton[1], V. Broadbent[4], A. Horwich[5], J. Levitt[3], T.J. McElwain[1], S.T. Meller[1], M. Mott[2], A. Oakhill[2] & J. Pritchard[3]

[1]*Department of Paediatric Oncology, The Royal Marsden Hospital, Downs Road, Sutton, Surrey; UK.* [2]*Royal Hospital for Sick Children, Bristol;* [3]*Hospital for Sick Children, Great Ormond Street, London;* [4]*Paediatric Department, Addenbrooke's Hospital, Cambridge; and* [5]*Department of Radiotherapy, Royal Marsden Hospital.*

**Summary** Between February 1986 and July 1988 a total of 21 children aged 1 to 16 years with malignant germ cell tumours (MGCT), 18 with either metastatic disease or unresectable primary tumour, received the JEB regimen – carboplatin dosage calculated from the EDTA glomerular filtration rate (approximately 600 mg m$^{-2}$), etoposide 120 mg m$^{-2}$ daily × 3, and bleomycin 15 mg m$^{-2}$ weekly. Primary sites were: testis (6), ovary (8), sacrococcyx (4), pineal gland (2) and vagina (1). AFP levels were elevated in 19, β-HCG in 8. Complete marker response was achieved in 19 out of 19 evaluable patients and complete remission of measurable tumour in 16 out of 19, 12 with chemotherapy alone and 4 with the addition of surgery. A reduction in glomerular filtration rate greater than 10% occurred in 3 of 12 evaluable patients; in none greater than 20%. Sequential audiography was normal in 11 out of 12 evaluated. The regimen was myelosuppressive with WHO grade III or IV myelosuppression occurring in 12 patients.

Three patients have relapsed; one with a pineal germinoma who relapsed in the abdomen six months after diagnosis, and two with sacrococcygeal teratomas and lung metastases. Two of these remain in second complete remission after further treatment. There was one death from probable bleomycin pulmonary toxicity. We conclude that this regimen is simple to administer and, apart from myelosuppression, it is well tolerated. It appears to have comparable efficacy to cisplatin-based regimens but with much less nephrotoxicity and ototoxicity and avoids the use of alkylating agents and anthracyclines.

Dramatic improvement in cure rates of adults with malignant germ cell tumours using cisplatin-based regimens have been mirrored in paediatric practice (Einhorn & Donohue, 1977; Peckham *et al.*, 1983; Williams *et al.*, 1987; Pinkerton *et al.*, 1986; Mann *et al.*, 1987). With short-duration chemotherapy, non-mutilating surgery and avoidance of radiotherapy or alkylating agents, the majority of children, including many with metastatic MGCT, can be cured. With such a favourable outcome emphasis is now placed on avoiding immediate and delayed toxicity. The PVB (cisplatin, vinblastine, bleomycin) or BEP (bleomycin, etoposide, cisplatin) regimens, without alkylating agents, probably avoid sterility and reduce second malignancy risks but are accompanied by significant renal and auditory toxicity. Even with careful hydration, mannitol and monitoring of glomerular filtration rate (GFR) by $^{51}$Cr-EDTA clearance, the majority of children will have a significant fall in GFR. Though hearing loss affects only high frequencies in most cases, it is still of clinical significance (Brock *et al.*, 1988). Although long-term studies are few (Brock *et al.*, 1988; Koliouskas *et al.*, 1985), there is little evidence that either renal function or hearing loss shows meaningful improvement with time.

Substitution for cisplatin by a less toxic analogue such as carboplatin has been shown to be effective in ovarian cancer (Calvert *et al.*, 1982; Wiltshaw *et al.*, 1985) and this drug has clear activity in adult Phase II studies with MGCT (Horwich *et al.*, 1988). There is, however, little or no evidence that significant non-cross resistance exists in cisplatin-resistant tumours and its use in the JEB regimen is primarily to avoid toxicity. In this regimen, carboplatin is given at a dose based on renal function calculated from the Calvert formula (Calvert *et al.*, 1989). This is predicted to produce an area under the drug concentration curve (AUC) which may lead to significant but manageable myelosuppression but possibly maximum therapeutic effect. Early studies in adults show clearly that inferior results are achieved with carboplatin unless the dose is pushed to myelosuppressive levels (Horwich *et al.*, 1989).

Correspondence: C.R. Pinkerton.
Received 29 January 1990; and in revised form 22 March 1990.

Etoposide and bleomycin are used as in the standard BEP regimen (Peckham *et al.*, 1983). There is some controversy about the necessity of weekly rather than 3-weekly bleomycin. In this study, if it was possible to monitor lung function, children were given this drug weekly. Infants, whose lung function could not be tested, received bleomycin 3-weekly after the first two cycles of chemotherapy.

### Patients and methods

Twenty-one children aged 1 to 16 years (median 11 years) received the JEB regimen. Nineteen were previously untreated and two had received single courses of PVB and BEP chemotherapy respectively. Clinical details are shown in Table I. Serum markers (α-fetoprotein and β-HCG) were estimated in all patients by immunoassay. Staging investigations included PA and lateral chest X-ray, CT chest scan, isotope bone scan and abdominal ultrasound or CT scan. Lymphography was not performed.

Indications for chemotherapy were the presence of metastatic disease in lung (6), lymph nodes (6), or peritoneum (1); bulky, unresectable primary tumour (5), peritoneal spill at surgery (3), or an intracranial primary (2). The testis was the primary site in 6 patients, ovary in 8 and sacrococcygeal area in 4. A one-year-old infant with a large vaginal tumour was treated electively with chemotherapy alone to avoid mutilating surgery. One boy with a pineal germinoma received JEB after complete resection and before irradiation. A second patient with a pineal tumour had undergone surgery but developed rapidly progressive disease within a month of this. α-FP was elevated in 19 patients, β-HCG in 8. In no patient was both α-FP and β-HCG normal. The chemotherapy is outlined in Figure 1. Bleomycin (15 mg m$^{-2}$) was given weekly as a slow intravenous infusion. Etoposide (120 mg m$^{-2}$) was administered daily × 3 as a 1 to 3 hour infusion and carboplatin infused over one hour. The formula for calculating the dose of carboplatin was based on the uncorrected $^{51}$Cr-EDTA clearance. In four children the GFR was not measured before treatment and doses based on surface area were given (400–500 mg m$^{-2}$). In four the carboplatin dose was given according to surface area despite the

GFR being known, and in three patients an AUC of less than 5 mg ml$^{-1}$ min$^{-1}$ was electively chosen by the physician. Tumour response was reassessed after 2 and 4 courses and CR documented using markers and imaging with X-ray or CT scan. Patients were given a minimum of four courses of JEB with additional cycles dependent on initial bulk of disease and the time to CR. Usually, chemotherapy was continued for 2 courses beyond CR.

**Table I** Clinical characteristics of patients

| Patient | Age at diagnosis (years) | Pathology[a] & serum markers | Sites of disease |
|---|---|---|---|
| 1 | 1 | Yolk sac<br>α-FP 4000 | Relapse in abdominal nodes 3/12 after testis primary resected |
| 2 | 11 | Mixed yolk sac & mature<br>α-FP 3000 | Ovary (resected with rupture) |
| 3 | 11 | Germinoma<br>α-FP 43 | Pineal mass; complete macroscopic resection |
| 4 | 15 | Yolk sac<br>α-FP 38 | Testis, (resected)<br>Para-aortic nodes |
| 5 | 2 | Yolk sac<br>α-FP 8855 | Sacrococcyx<br>Lung |
| 6 | 13 | Dysgerminoma<br>β-HCG 99 | Ovary (resected with rupture). Para-aortic nodes. |
| 7 | 15 | Immature & choriocarcinoma<br>α-FP 115<br>β-HCG 438 | Testis, (resected)<br>Lung and para-aortic nodes |
| 8 | 13 | Yolk sac choriocarcinoma<br>α-FP 7000<br>β-HCG 113000 | Ovary<br>Lung |
| 9 | 16 | Yolk sac & mature<br>α-FP 1340 | Ovary (resected)<br>Masses R. ovary & peritoneum |
| 10 | 11 | Yolk sac & mature<br>α-FP 45 | Testis, para-aortic nodes |
| 11 | 1 | Yolk sac<br>α-FP 545 | Vagina |
| 12 | 16 | Yolk sac<br>α-FP 155<br>β-HCG 592 | Testis (resected)<br>Bilateral lung 2° |
| 13 | (birth) | No Biopsy<br>α-FP 23820 | Lung 2° 13/12 after removal of 'benign' sacrococcygeal teratoma |
| 14 | 9 | Yolk sac<br>α-FP 2015<br>β-HCG 1455 | Ovary resected with rupture |
| 15 | 2½ | No Biopsy<br>α-FP 103000 | Sacrococcyx<br>Lung 2° |
| 16 | 14 | Immature<br>α-FP 7300 | Ovary |
| 17 | 12½ | Dysgerminoma<br>β-HCG 490 | Ovary |
| 18 | 16 | Immature<br>α-FP 25<br>β-HCG 13 | Testis, (resected)<br>Para-aortic nodes |
| 19 | 8 | No Biopsy<br>α-FP 1500<br>(CSF 2400) | Pineal |
| 20 | 17½ | Yolk sac<br>α-FP 291000 | Ovary |
| 21 | 2 | Yolk sac<br>α-FP 66000<br>β-HCG 105 | Sacrococcyx |

[a]Dehner classification (Dehner, 1986).

| Day: | 1 | 2 | 3 | 9 | 12 |
|---|---|---|---|---|---|
| JM8[a] (Carboplatin) | ↓ | | | | |
| Bleomycin 15 mg m$^{-2}$ I.V. | | ↓ | | ↓ | ↓ |
| Etoposide 120 mg m$^{-2}$ I.V. | ↓ | ↓ | ↓ | | |

[a]Dose mg = ([EDTA uncorrected × 1.2] + 20) × AUC (6).

**Figure 1** Outline of JEB chemotherapy. Each course was repeated at 21-day intervals provided the neutrophil count $> 1.0 \times 10^9 l^{-1}$ and platelets $> 100 \times 10^9 l^{-1}$.

### *Monitoring Toxicity*

Estimates of plasma urea and creatinine have been shown to be inaccurate indicators of renal impairment in children receiving cisplatin (Womer et al., 1985). $^{51}$Cr-EDTA GFR was therefore estimated before treatment and repeated during treatment in most patients. Formal audiometry was done during and following treatment in patients old enough to co-operate. The hearing was graded according to a scale devised specifically to quantify cisplatin-related ototoxicity in children (Brock et al., 1988). In patients who could co-operate, spirometric respiratory function tests and CO diffusion tests were performed at intervals during and following treatment.

### Results

### *Tumour response and outcome*

The total number of courses given ranged from 4 to 6 (median 4). Marker CR was achieved in all patients after between two and four courses of chemotherapy. The estimated serum α-FP t½ ranged from 3 to 12 days (median 7 days) and of β-HCG from 3 to 15 days (median 4) (Table III). One boy with a pineal germinoma (case No 3), who had no measurable disease following initial surgery, developed progressive abdominal disease with multiple peritoneal seedlings, probably related to a ventriculo-peritoneal shunt. Despite cranio-spinal irradiation and introduction of an intensive cisplatin-containing regimen he failed to respond. Patient 5 with a sacrococcygeal primary and lung metastases, achieved rapid CR but tumour recurred at the primary site 4 months after completion of treatment. Coccyxectomy had not initially been performed electively because of radiological CR at primary and metastatic sites. Patient 16 developed recurrent lung metastases 6 months after having achieved CR.

With chemotherapy alone complete remission of disease on X-ray or CT scan occurred in 12 of 18 evaluable patients; residual primary disease was completely resected in 4 cases; 2 showed active tumour and 2 mature teratoma (Table IV). 'Second look' surgery was performed in 6 patients with no imageable disease and confirmed clinical CR in each.

Minor residual abnormalities were seen on CT scan in three patients but secondary surgery was not performed. These abnormalities were in the abdomen in two cases and in the lung in a third child. All abnormal CT images subsequently resolved and the patients remain free from disease. One patient (number 10), developed progressive peritoneal disease on CT scan during chemotherapy despite normalization of α-FP levels. At laparotomy, the tumour was resected and shown to consist entirely of differentiated teratoma. No further treatment was given and she remains free from disease 15 months from diagnosis.

Hearing was not evaluated in 7 patients because of their young age. In one of 12 patients, adequately evaluated, there was evidence of high tone hearing loss during treatment (20 dB loss at 4 KHz), but this returned to normal 3 months later. $^{51}$Cr-EDTA clearance declined >10% from the original value in 3 of 12 patients adequately studied; 12, 15 and 20% respectively. (See Table II).

**Table II** Number of JEB courses, toxicity and estimated AUC of carboplatin

| No. | Number of courses given | Carboplatin AUC (mg ml$^{-1}$ min) | $^{51}$Cr EDTA clearance (ml min$^{-1}$ 1.73 m$^{-2}$) (JEB course) 1 | 2 | 3 | 4 | 5 | 6 | Haematological toxicity (WHO grade) |
|---|---|---|---|---|---|---|---|---|---|
| 1 | 4 | 5 | 112 | . | 90 | 117 | 109 | . | Gd 4 neut & tcp |
| 2 | 5 | 5 | 103 | 114 | . | 113 | . | 116 | Gd 3 neut; Gd 2 tcp |
| 3 | 4 | 5 | 99 | . | 106 | 147 | . | . | None |
| 4 | 4 (+ BEP × 1) | 3 (400 mg m$^2$)* | 132 | . | . | . | 169 | . | Not documented |
| 5 | 5 | 4.5 | 135 | . | . | 107 | . | 163 | Gd 4 tcp |
| 6 | 6 | 6 | 107 | . | . | 124 | . | 129 | Gd 1 tcp<br>Gd 4 neut |
| 7 | 4 | 5 | 140 | 140 | 140 | . | . | . | Gd 1 tcp<br>Gd 3 neut |
| 8 | 4 | 4 (600 mg m$^2$)* | 194 | . | 219 | . | . | . | Not documented |
| 9 | 5 | 6 | 115 | . | 140 | 118 | . | . | Gd 3 neut<br>Gd 2 tcp |
| 10 | 4 | 5 | 148 | 130 | . | 125 | . | . | Gd 2 neut<br>Gd 1 tcp |
| 11 | 4 | 6 | 121 | 124 | 97 | . | 97 | . | Gd 4 neut & tcp<br>(Gd 4 sepsis) |
| 12 | 6 | 4.5 | 142 | . | 129 | . | 125 | . | Gd 1 tcp |
| 13 | 6 | (400 mg m$^2$) | . | . | . | . | . | . | Gd 1 tcp<br>Gd 2 neut |
| 14 | 6 | 4 (500 mg m$^2$) | 112 | . | . | . | . | . | Gd 4 tcp<br>Gd 3 neut |
| 15 | 5 | (400 mg m$^2$) | . | . | . | . | . | . | Gd 4 neut<br>(Gd 3 sepsis) |
| 16 | 4 | (500 mg m$^2$) | . | . | . | . | . | . | Not documented |
| 17 | 5 | 6.5 (500 mg m$^2$) | 63 | . | . | . | . | . | Gd neut<br>Gd 4 tcp<br>(Gd 4 sepsis) |
| 18 | 4 | 4 | 138 | . | . | . | . | . | Gd 3 tcp<br>Gd 4 neut |
| 19 | 4 | (500 mg m$^2$) | . | . | 170 | . | . | . | Not documented |
| 20 | 2 | 6 | 112 | . | . | . | . | . | Not documented |
| 21 | 4 (+ BEP × 1) | 6 | 129 | . | . | . | . | . | Gd 4 tcp |

neut = neutropenia: tcp = thrombocytopenia *( ) = dose given

As expected, haematological toxicity was comparatively severe with over half the patients developing WHO grade III or IV myelosuppression on one or more occasions. Platelet transfusions were required in only two patients but three required parenteral antibiotics for fever during neutropenic periods. Chemotherapy was given on time in most patients with a median interval between courses of 24 days (range 21–45).

Lung function tests were abnormal in two patients. One girl (case No 2) had a pre-existing restrictive defect which persisted but did not worsen. One boy (case No 4) developed radiological evidence of early bleomycin toxicity with co-existing alterations in lung function tests. This was treated with steroids and has resolved. Case No 14, a 16-month-old girl, died with an acute pneumonitis 3 months after receiving 6 courses of JEB. No pathogen was identified and although no autopsy was performed bleomycin appeared to be implicated. She had received weekly bleomycin with each course of JEB.

### Discussion

This pilot study demonstrates both the efficacy and the tolerability of substituting carboplatin for cisplatin, combined with etoposide and bleomycin, in paediatric MGCT. In this regimen, a carboplatin AUC of 5–6 mg ml$^{-1}$ min, which in children with normal renal function is equivalent to approximately 600 mg m$^{-2}$, was recommended. This is somewhat higher than the AUC recommended in adults (4–5) which is predicted to result in moderately severe thrombocytopenia (Calvert *et al.*, 1989). As a single agent, at a dose of 400 mg m$^{-2}$, carboplatin has produced high response rates in untreated patients with localized seminomatous and non-seminomatous germ cell tumours (NSGCT) (Horwich *et al.*, 1988). In one study using JEB in poor risk NSGCT in adults only 40% achieved CR with chemotherapy and surgery (Motzer *et al.*, 1987). In that study, however, the dose of carboplatin was only 350 mg m$^{-2}$ which would yield an AUC in the range of 2–3 mg ml$^{-1}$ min. With a higher dose of

**Table III** Clinical and tumour marker response and outcome

| *No.* | *Time to marker remission*<br>*Half-life* | *Time to clinical response*<br>*Evaluation of response* | *Outcome* | *Months after diagnosis* |
|---|---|---|---|---|
| 1 | CR 2 courses<br>α-FP 6d | CR 4 courses<br>No tumour at surgery | NED | 31 |
| 2 | CR 2 courses<br>α-FP 5d | Non evaluable | NED | 33 |
| 3 | CR 2 courses<br>α-FP 12d | Non evaluable | Died<br>Relapsed in peritoneum (6/12) | 9 |
| 4 | CR 4 courses | Residual lung CT 'abnormalities'<br>PR → CR with surgery<br>'active tumour' | NED | 38 |
| 5 | CR 3 courses<br>α-FP 6d | CR 5 courses<br>CT scan | Relapsed at primary (4/12)<br>Resected → CR2 | 17 |
| 6 | CR 2 courses<br>α-FP 3d | CR 6 courses<br>CT scan | NED | 23 |
| 7 | NE* | Lung CR 3 courses<br>Minimal CT abnormality in abdomen. → CR with surgery<br>'mature teratoma' | NED | 21 |
| 8 | NE* | PR → CR<br>with surgery<br>'active tumour' | NED | 15 |
| 9 | CR 3 courses<br>α-FP 8d | NR → CR<br>with surgery<br>'mature teratoma' | NED | 15 |
| 10 | CR 1 course<br>α-FP 8d | 'CR' 4 courses<br>Minimal CT abnormality | NED | 13 |
| 11 | CR 2 courses<br>α-FP 8d | CR 4 courses<br>No tumour at surgery | NED | 10 |
| 12 | CR 2 courses<br>α-FP 8d | 'CR' 6 courses<br>Minimal CT abnormality | NED | 19 |
| 13 | CR 2 courses<br>α-FP 8d | CR 1 course<br>CT scan | Died.<br>Bleomcyin lung | 5 |
| 14 | CR 2 courses<br>HCG 4d<br>α-FP 7d | Non evaluable | NED | 15 |
| 15 | CR 5 courses<br>α-FP 10d | CR 3 courses<br>CT scan<br>No tumour at surgery | Lung relapse (6/12)<br>CR 2 after surg.<br>IF, Epi, VCR | 17 |
| 16 | CR 2 courses<br>α-FP 5d | CR 2 courses<br>CT scan | NED | 41 |
| 17 | CR 3 courses<br>HCG 15d | CR 3 courses<br>No tumour at surgery | NED | 11 |
| 18 | CR 1 course<br>α-FP 6d<br>HCG 3d | CR 4 cycles<br>CT scan | NED | 9 |
| 19 | CR 2 courses<br>α-FP 7d | CR 2 courses<br>CT scan | NED | 12 |
| 20 | CR 3 courses<br>α-FP NE | CR 2 courses<br>No tumour at surgery | NED | 20 |
| 21 | CR 4 courses<br>α-FE NE | CR 4 courses<br>No tumour at surgery | NED | 11 |

NED = no evidence of disease. *inadequate data

**Table IV** Response to chemotherapy in extra-cranial tumours

N = 19 —— JEB alone

- 2 not evaluable (Rupture at surgery)

- CR 11 (surgically proven in 6)

  - 1 toxic death

  - 2 Relapsed → 2

  - 8 NED 9–31 mths

- Minor CT Residue (2 abdomen, *1 lung) —— Remain NED 13, 19, *38 mths.

- CT Residue → Surgery

  - 2 Active Disease NED 15, *38

  - 2 Mature Tumour NED 15, 21

(* same patient)

NED = no evidence of disease carboplatin and an estimated AUC of over 4 mg ml$^{-1}$ min$^{-1}$, 39 out of 39 previously untreated metastatic NSGCT patients achieved complete remission (Horwich *et al.*, 1989). Because of concern about compromising antitumour activity an AUC of 5–6 was recommended in this study. Comparatively severe myelosuppression was expected and accepted as a trade-off against reduced renal and auditory toxicity. Formal randomised studies comparing the regimen against PVB or BEP are in progress in adults.

This study confirms that carboplatin is not associated with the ototoxicity seen with the BEP regimen. There was a small decline in GFR in a minority of children but in adults this has been clearly shown to be transient (Hardy *et al.*, 1990).

The role of bleomycin has not been addressed in this study but major reservations have been expressed in the past about weekly administration of this drug. Deaths from respiratory failure have been reported in children receiving PVB (Mann *et al.*, 1987) and although other contributory factors may have been involved, there is some reason for concern. A link between cisplatin nephrotoxicity and bleomycin toxicity has been suggested (Dalgleish *et al.*, 1984). It is of note that one patient in this series developed clear evidence of lung toxicity although renal function remained normal after carboplatin. A small non-randomised study in adults has suggested that a reduced dose of bleomycin adversely affects the efficacy of BEP, but this was only in patients where the etoposide dose was also suboptimal (Brada *et al.*, 1987). By contrast, with full dose etoposide and cisplatin, the results were impressive despite the reduction to 3-weekly bleomycin. Current studies of BEP against EP in small bulk disease show little difference (Levi *et al.*, 1986; Stoter *et al.*, 1987) but the question has not been settled in high-risk patients. Limiting weekly bleomycin to the first two cycles in patients who cannot have lung function tests done is an option, or alternatively, only monthly treatment could be given in all patients. At present, there is a reluctance to omit the drug altogether in children, but once the results of randomised studies in adults are available this may be indicated. With the JEB regimen the response rate is comparable to that in children receiving cisplatin based regimens, with or without the addition of alkylating agents (Pinkerton *et al.*, 1986, Flamant *et al.*, 1984). With extra-cranial primaries a CR rate of 65% with chemotherapy alone, and 84% with chemotherapy plus surgery was achieved. All three patients not achieving CR have had minimal CT abnormalities which have remained unchanged, or resolved.

The role of surgery in gonadal disease is comparatively clear and is a useful way to confirm CR after chemotherapy or to resect any residual active disease. In the case of sacrococcygeal disease the necessity of coccyxectomy after radiological CR has been questioned. This procedure is however justified as an elective measure because of the difficulty in being sure that there is no residual tumour in this complex bony structure. The operation should be safe and without sequelae. In one child in the present series (No 5) disease recurred locally in the coccyx despite apparent CR.

Radiotherapy has little role in paediatric MGCT except in the event of refractory or relapsed disease. It continues to be used in pineal tumours because of reservations about the reliability of drug access to the CNS. It is becoming clear that this concern may be unfounded in some cases (Rustin *et al.*, 1989). There are, however, no reports yet of large series where radiotherapy was not given electively. It is likely that information will be gained from small infants in whom irradiation is omitted owing to concern about late sequelae.

Although there were no patients with very bulky mediastinal primaries or bone marrow or bone metastases, this was a comparatively high risk group and included six children with lung metastases and six with nodal disease. The risk categories devised for adult MGCT cannot readily be applied to paediatric patients. The absolute peak serum α-FP or β-HCG are not of the same significance as in adults provided levels decline within a relatively short half-life. 'Bulk disease' is difficult to define in the small child because the absolute tumour volume may be of more importance than volume in relation to the child's size. In adults, extragonadal tumours, mediastinal tumours in particular, continue to pose a therapeutic problem (Logothetis *et al.*, 1985). This may be a consequence of bulk disease or difficulty achieving complete resection of residual disease. It is of note that prognosis appears better with pure yolk sac histology in this subgroup. It is likely that JEB will not be sufficient for some patients and alternative strategies such as high-dose platinum and etoposide or high-dose intensity regimens such as the BEP/BOP or POMB/ACE may be necessary (Horwich *et al.*, 1989; Newlands *et al.*, 1983). Evaluation of the JEB regimen in unselected MGCT patients on a multicentre basis is currently underway by the United Kingdom Children Cancer Study Group. This study may help to define if there is a high risk group of patients in whom more toxic chemotherapy is justifiable.

J. Pritchard acknowledges financial support from the Imperial Cancer Research Fund and C.R. Pinkerton from the Leukaemia Research Fund and Sterling Oncology. T.J. McElwain is supported by the Cancer Research Campaign and the Medical Research Council. We are grateful to Tereza Gladwell for help with preparation of the manuscript.

**References**

BRADA, M., HORWICH, A. & PECKHAM, M.J. (1987). Treatment of favourable-prognosis nonseminomatous testicular germ cell tumors with etoposide, cisplatin, and reduced dose of bleomycin. *Cancer Treat. Rep.*, **71**, 655.

BROCK, P., PRITCHARD, J., BELLMAN, S. & PINKERTON, C.R. (1988). Ototoxicity of high-dose cis-platinum in children. *Med. Ped. Oncol.*, **16**, 368.

CALVERT, A.H., HARLAND, S.J., NEWELL, D.R. & 9 others (1982). Early clinical study of the cisdiamine 1,1-cyclobutanedicarboxylate platinum II. *Cancer Chemother. Pharmac.*, **9**, 140.

CALVERT, A.H., NEWELL, D.R., GUMBRELL, L.A., BOXALL, F.E., EELES, R.A. & HORWICH, A. (1989). The clinical pharmacokinetics of carboplatin: Prospective validation of a dosage formula for use in high dose single agent and combination studies. *Proc. ASCO*, **8**, 70 (abstr. 271).

DALGLEISH, A.G., WOODS, R.L. & LEVI, J.A. (1984). Bleomycin pulmonary toxicity. Its relation to renal dysfunction. *Med. Ped. Oncol.*, **12**, 313.

DENHER, L.P. (1986). Gonadal and extragonadal germ cell neoplasms-teratomas in childhood. In: Finegold, M. & Benington, J.L. (eds): *Pathology of Neoplasia in Children and Adolescents. Major Problems in Pathology*. W.B. Saunders: Philadelphia, **18**, 282.

EINHORN, L.H. & DONOHUE, J.P. (1977). Cisdiamminedichloroplatinum, vinblastine and bleomycin combination chemotherapy in disseminated testicular cancer. *Ann. Intern. Med.*, **87**, 293.

FLAMANT, F., SCWARTZ, L., DELONS, E., CAILLAUD, J.M., HARTMANN, O. & LEMERLE, J. (1984). Nonseminomatous malignant germ cell tumours in children. *Cancer*, **54**, 1687.

HARDY, J.R., TAN, S., FRYATT, I. & WILTSHAW, E. (1990). How nephrotoxic is carboplatin? *Br. J. Cancer*. (In press).

HARLAND, S. & HORWICH, A. (1987). What dose of carboplatin can be combined with etoposide and bleomycin in patients with testicular cancer? *Proc. ASCO*, **6**, 48 (abstr. 184).

HORWICH, A., DUCHESNE, G., DEARNALEY, D. & PECKHAM, M. (1988). Single agent carboplatin (SAC) as initial therapy for advanced seminoma. *Proc. ASCO*, **7**, 117 (abstr. 452).

HORWICH, A., DEARNALEY, D., HARLAND, S., PECKHAM, M.J. & HENDRY, W.F. (1989). Carboplatin etoposide bleomycin (CEB) combination chemotherapy is effective in good prognosis metastatic testicular non seminomatous germ cell tumours (NSGCT). *Proc. ASCO*, **8**, 134 (abstr. 521).

HORWICH, A., BRADA, M., NICHOLLS, J. & 4 others (1989). Intensive induction chemotherapy for poor risk non-seminomatous germ cell tumours. *Eur. J. Cancer Clin. Oncol.*, **25**, 177.

KOLIOUSKAS, D., BARRATT, T.M., CRAFT, A.W. & 4 others (1985). Is there recovery of renal function after cisplatin therapy? *Proc. SIOP*, 301.

LEVI, J., RAGHAVAN, D., HARVEY, V. & 5 others (1986). Deletion of bleomycin from therapy for good prognosis advanced testicular cancer. *Proc. ASCO*, **5**, 97 (abstr. 374).

LOGOTHETIS, J., SAMUELS, M.L., SELIG, D.E. & 4 others (1985). Chemotherapy of extragonadal germ cell tumors. *J. Clin. Oncol.*, **3**, 316.

MANN, J.R., PEARSON, D., BARRETT, A., RAAFAT, F., BARNES, J.M. & WALLENDSZUS, K.R. (1987). UKCCSG Malignant germ cell tumours – treatment results. *Proc. SIOP*, 57.

MOTZER, R.J., BOSL, G.J., YAGODA, A. & GOLBEY, R. (1987). Treatment of poor risk nonseminomatous germ cell tumor patients with carboplatin + etoposide + bleomycin. *Proc. AACR*, **28**, 202 (abstr. 804).

NEWLANDS, E.S., BEGENT, R.H.J., RUSTIN, G.J.S., PARKER, D. & BAGSHAWE, K.D. (1983). Further advances in the management of malignant teratomas of the testis and other sites. *Lancet*, **i**, 948.

PECKHAM, M.J., BARRETT, A., LIEW, K.H. & 5 others (1983). The treatment of metastatic germ cell testicular tumours with bleomycin, etoposide and cisplatin (BEP). *Br. J. Cancer*, **47**, 613.

PINKERTON, C.R., PRITCHARD, J. & SPITZ, L. (1986). High complete response rate in children with advanced germ cell tumors using cisplatin-containing combination chemotherapy. *J. Clin. Oncol.*, **4**, 194.

RUSTIN, G.J.S., NEWLANDS, E.S., BEGENT, R.H.J., DENT, J., BAGSHAWE, K.D. (1989). Weekly alternating etoposide, methotrexate, and actinomycin/vincristine and cyclophosphamide chemotherapy for the treatment of CNS metastases of choriocarcinoma. *J. Clin. Oncol.*, **7**, 900.

STOTER, G., KAYE, S., JONES, W. & 8 others (1987). Cisplatin and VP16 + bleomycin in good risk patients with disseminated non-seminomatous testicular cancer; results of a randomized EORTC GU group study. *Proc. ECCO*, **49**, (abstr. 681).

WILLIAMS, S.D., BIRCH, R., EINHORN, L.H., IRWIN, L., GRECO, F.A. & LOEHRER, P.J. (1987). Treatment of disseminated germ-cell tumors with cisplatin, bleomycin and either vinblastine or etoposide. *N. Engl. J. Med.*, **316**, 1435.

WILTSHAW, E., EVANS, B. & HARLAND, S. (1985). Phase III randomised trial of cisplatin versus JM8 (carboplatin) in 112 ovarian cancer patients, stages III and IV. *Proc. ASCO*, **4**, 121 (abstr. C 471).

WOMER, R.B., PRITCHARD, J. & BARRATT, T.M. (1985). Renal toxicity of cisplatin in children. *J. Pediatr.*, **106**, 659.